# Validating Questionnaires for Lower Limb Rehabilitation Systems and Devices: A Scoping Review

**DOI:** 10.3390/sports13010004

**Published:** 2025-01-02

**Authors:** Angie D. Moscoso, Vera Z. Pérez, Manuel J. Betancur

**Affiliations:** Facultad de Ingeniería Electrónica, Universidad Pontificia Bolivariana, Medellín 050031, Colombia; angie.moscoso@upb.edu.co (A.D.M.); manuel.betancur@upb.edu.co (M.J.B.)

**Keywords:** validating questionnaires, exergames, lower limbs, rehabilitation

## Abstract

This article aims to make a scoping review of Validating Questionnaires used in the field of lower limb (LL) rehabilitation in which systems, devices or exergames are used. Its main objective is to provide a more comprehensive understanding of the results obtained in the validation of questionnaires, as well as to identify specific criteria for evaluating systems, devices or exergames in the area of LL rehabilitation, through the analysis of validating instruments and their application in different associated contexts. The article details the methodology employed, a PRISMA ScR method review which included database research and an evaluation of the selected studies. Inclusion and exclusion criteria were applied to select all relevant studies, resulting in 81 studies after initial review based on titles and abstracts. Subsequently, the criteria were again applied to read the full text, resulting in 58 final studies. The document distinguishes between standardized and non-standardized validating questionnaires, emphasizing that standardized validating questionnaires have undergone rigorous statistical processes to ensure their validity, reliability and consistency. The information compiled in the tables provides a solid basis for identifying and evaluating validation questionnaires in the above-mentioned context. This resource constitutes an accurate and reliable reference for selecting the most appropriate instruments for future research and comparisons with similar work. This article is a valuable resource for those interested in the validation of questionnaires used in the field of lower limb rehabilitation systems/devices/exergames.

## 1. Introduction

According to the last report of World Health Organization, there are about 1.3 billion people with disabilities, about 16% of the world’s population [1]. Rehabilitation plays a crucial role in helping people recover or improve their functional capabilities after an injury, illness or health problem. The need for rehabilitation worldwide is substantial, as by the year 2024, it was estimated that 2.4 billion people around the world may benefit from these services [2]. Musculoskeletal disorders, sensory impairment and injuries are the main causes of disability that require rehabilitation. Musculoskeletal disorders affect approximately 1.710 million people (22%), while sensory impairments affect about 730 million (9.4%). Injuries, including spinal cord injuries and traumatic brain injuries, affect approximately 1000 million people (19%) [3,4,5].

Rehabilitation offers numerous benefits, such as improved functional capabilities, reduced pain and suffering, and improved overall life quality. It also plays a vital role in helping people regain their functional abilities and improve their well-being.

There are several modalities to carry out the rehabilitation process. According to the form of rehabilitation that can be incorporated, a rehabilitation system, refers to a set of tools, devices, or structured programs designed to help individuals recover or improve physical, cognitive, or emotional abilities lost due to injury, illness, or disability. Rehabilitation systems can include traditional therapeutic practices, as well as advanced technologies like robotic devices, virtual reality, or assistive software tailored to meet individual patient needs, used in conventional therapy or telemedicine. Besides those, another form that has emerged with new technologies is the rehabilitation with Exergames which refers to video games that require body movements or physical effort of the players to interact with the game environment and control the game mechanics, in this case, the potential applications of Exergames extend beyond the promotion of physical activity and health [6]. Studies have shown significant promise in therapeutic and rehabilitation settings, where motor skills training, balance improvement, and cognitive rehabilitation can be aided [7]. When talking about systems that use exergame in rehabilitation, it is essential to understand that, like other systems, they must have robustness and functionality characteristics to fulfill their purpose. However, it is also vital that these devices are attractive, motivating, and entertaining for patients [8]. In this regard, to determine the quality and impact of these exergame systems used in rehabilitation, the use of fundamental tools such as validation questionnaires plays a crucial role in collecting data on effectiveness, usability, and perceived user satisfaction. A validation questionnaire is a tool used to assess the reliability, validity, and usability of a specific instrument (e.g., a survey or test) in measuring a particular construct or outcome. Validation ensures that the questionnaire accurately reflects the intended attributes and performs consistently across different populations or settings. Through these instruments, valuable information is obtained that permits to objectively evaluate the performance of exergame systems in the context of rehabilitation, taking into account both standardized and non-standardized validating questionnaires to assess the system based on its evaluation purpose [9]. Ultimately, this combination of exergame and validating questionnaires strengthens the scientific basis of rehabilitation, ensuring that the devices used are effective, safe and capable of providing the best results in patient recovery.

After developing a prototype it is important to define and review whether it has the potential to be successful when used on people and one of the ways to verify this and move forward in the TRL process is through technical testing and people testing with validating questionnaires. This is because if we consider the process of interaction between the user and the equipment, the user serves as a measuring element of the functionality of the equipment and one of the mechanisms to evaluate this functionality has been the validating questionnaires.

The need for a literature review is evidenced by the fact that there is a wide variety of questionnaires in the literature and that no specific selection criteria have been identified for the choice of any of them for a particular type of rehabilitation system evaluation. There is no clear understanding of which validating questionnaires are best applicable according to the specific context of the systems developed and therefore a need has been identified to systematically review the existing literature on the subject associated with upper and lower limb rehabilitation and to build the necessary documentation that will allow future research in the area to apply the necessary questionnaires according to their specific system.

Although in some investigations the authors choose to use Ad-hoc questionnaires, in others, researchers opt to use validating questionnaires in which content, criterion, construct, concurrent, predictive, discrimination, internal consistency and reliability validity have been taken into account [10,11].

In this framework, the article, as study objectives, aims to report in a systematized way the validation criteria, the validation instruments by criteria with their measurement scale interpretation and several examples of their application. Section 2 presents the research methodology and the filtering of the information. Section 2 presents the research methodology and the filtering of the information. Section 3 presents the results in a systematized way, taking into account the validation criteria, the validating instruments with their scale measurement interpretation and the most relevant studies found in the literature. Section 4 presents a discussion of the results and Section 5 draws a conclusion regarding the subject matter covered.

## 2. Methodology

### 2.1. Study Design

A scoping review was conducted according to the Preferred Reporting Items for Systematic Reviews and Meta-Analyses for Scoping Reviews (PRISMA-ScR) criteria (see Supplementary Materials) [12]. The scoping review process included several co-creation exercises among the authors to define the study, formulate questions, refine ideas, develop a working plan, review progress, discuss findings, and outline content for different sections. Additionally, detailed individual processes were required to identify, select, and synthesize studies related to validation instruments for evaluating systems, devices, or exergames in lower limb rehabilitation. Key stages of the review process are explicitly documented in compliance with PRISMA standards. No formal registration was performed for this review.

**Figure 1:**
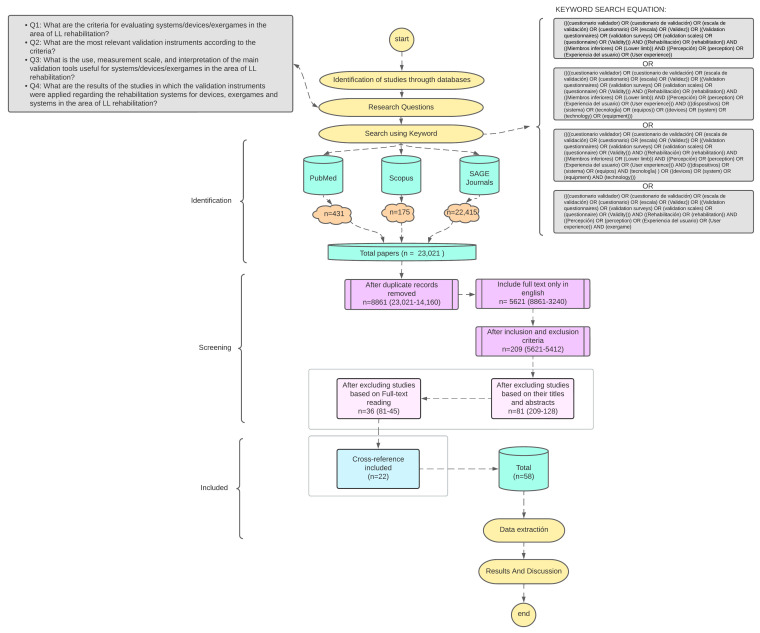
Methodology flowchart.

Figure 1 shows the flow diagram corresponding to the method used in this scoping review.

### 2.2. Research Questions

The study’s research questions were designed through a rigorous co-creation process to directly address its objectives. Each question reflects a specific aspect of the study objectives.

Initially, based on the recent challenges faced by V.Z.P. and M.J.B in research projects on rehabilitation, the need to explore more thoroughly adequate instruments to measure performance in systems, devices, or exergames was identified. An initial co-creation exercise among the three authors resulted in a matrix of information containing preliminary ideas about specific topics to explore, known results, and potential methodologies for conducting the scoping review. At the conclusion of the co-creation exercise, research questions were addressed to guide and orient the search for validation questionnaires in the area of lower limb (LL) rehabilitation. The research questions were structured in a particular order, from general criteria to specific applications, to address the study´s objectives progressively, starting from identifying criteria (Q1, Q2) to exploring their applications and results (Q3, Q4), as follows:

- Q1: What are the criteria for evaluating systems/devices/exergames in the area of LL rehabilitation?
- Q2: What are the most relevant validation instruments according to the criteria?
- Q3: What is the use, measurement scale, and interpretation of the main validation tools useful for systems/devices/exergames in the area of LL rehabilitation?
- Q4: What are the results of the studies in which the validation instruments were applied regarding the rehabilitation systems for devices, exergames and systems in the area of LL rehabilitation?

Question Q1 is oriented and contributes to identifying specific criteria for evaluating systems, devices, and exergames in LL rehabilitation, the first study objective. Question Q2 is oriented toward finding the most relevant validation instruments to the citeria. In addition, questions Q3 seek to understand the use, measurement scale, and interpretation of validation instruments in the context of LL rehabilitation. These two questions are aligned with the second study objective. Finally, Q4 analyzes the results of previous research that applied validation instruments to specific systems, devices, or exergames used in this area of rehabilitation. It is aligned with the third study objective.

### 2.3. Information Sources

An exhaustive bibliographic search was conducted in electronic databases, such as PubMed, Scopus, and Sage Journal, which offer extensive coverage of scientific and medical journals, conferences, books and patents. PubMed was chosen for its focus on high-quality, peer-reviewed life sciences and biomedical articles, which are central to our research. Scopus was included due to its broad coverage of scientific disciplines, including engineering and technology, and its inclusion of conference proceedings and patents, which are essential for capturing cutting-edge developments in the field. Finally, Sage Journal was selected for its interdisciplinary approach, providing access to journals in both health and social sciences, which allows for a broader exploration of the subject matter. These three databases together offer a comprehensive range of resources, ensuring that our study is based on reliable, diverse, and up-to-date information.

### 2.4. Search Strategy

Four search equations were used in each database, where the following combination of keywords was used with the Boolean operators AND and OR:

- (((cuestionario validador) OR (cuestionario de validación) OR (escala de validación) OR (cuestionario) OR (escala) OR (Validez)) OR ((Validation questionnaires) OR (validation surveys) OR (validation scales) OR (questionnaire) OR (Validity))) AND ((Rehabilitación) OR (rehabilitation)) AND ((Miembros inferiores) OR (Lower limb)) AND ((Percepción) OR (perception) OR (Experiencia del usuario) OR (User experience)).
- ((((cuestionario validador) OR (cuestionario de validación) OR (escala de validación) OR (cuestionario) OR (escala) OR (Validez)) OR ((Validation questionnaires) OR (validation surveys) OR (validation scales) OR (questionnaire) OR (Validity))) AND ((Rehabilitación) OR (rehabilitation)) AND ((Miembros inferiores) OR (Lower limb)) AND ((Percepción) OR (perception) OR (Experiencia del usuario) OR (User experience))) AND (((dispositivos) OR (sistema) OR (tecnología) OR (equipos)) OR ((devices) OR (system) OR (technology) OR (equipment))).
- ((((cuestionario validador) OR (cuestionario de validación) OR (escala de validación) OR (cuestionario) OR (escala) OR (Validez)) OR ((Validation questionnaires) OR (validation surveys) OR (validation scales) OR (questionnaire) OR (Validity))) AND ((Rehabilitación) OR (rehabilitation)) AND ((Miembros inferiores) OR (Lower limb)) AND ((Percepción) OR (perception) OR (Experiencia del usuario) OR (User experience))) AND (((dispositivos) OR (sistema) OR (equipos) AND (tecnología) ) OR ((devices) OR (system) OR (equipment) AND (technology))).
- (((cuestionario validador) OR (cuestionario de validación) OR (escala de validación) OR (cuestionario) OR (escala) OR (Validez)) OR ((Validation questionnaires) OR (validation surveys) OR (validation scales) OR (questionnaire) OR (Validity))) AND ((Rehabilitación) OR (rehabilitation)) AND ((Percepción) OR (perception) OR (Experiencia del usuario) OR (User experience)) AND (exergame).

### 2.5. Elegibility Criteria

After the conceptual and strategic design of the study, the formulation of research questions, and the definition of objectives, inclusion and exclusion criteria were applied based on the PCC framework to ensure relevance and clarity, while focuses on exploring and mapping existing research. The eligibility criteria, outlined in Table 1, are related to the area of study and address the defined research questions.

Additionally, filters are applied to include only full-text studies in English.

### 2.6. Selection of Sources of Evidence

The first version of the search strategy was developed by A.D.M., and a complete analysis of these results was conducted by all authors in a co-creation session. Finally, V.Z.P. created a second version, updating the tables with new information considered important by the authors during the co-creation session, returning to the original sources. The first version consisted of three main stages: identification, selection, and inclusion. The identification stage involved performing an advanced search in the selected databases using the key terms and Boolean operators presented in Section 2.3, combining the four search equations with the OR operator. In the selection stage, duplicate documents from the three databases were eliminated. After obtaining the total number of documents, full-text filters were applied to include only English-language documents, and the results were imported into Mendeley (Version 1.19.8) for easier management and selection. Subsequently, the inclusion and exclusion criteria defined in Table 1 were applied, and the remaining documents were reviewed to exclude those that did not meet the criteria based on their titles, abstracts, and full text. Finally, in the inclusion stage, after a complete reading of the documents, relevant studies that met the criteria and were related to the research topic were included. The information from the remaining articles was recorded in tables created in Microsoft Word Professional Plus 2019.

Subsequently, all authors reviewed the information in the tables and discussed the ideas they considered useful for the discussion section. This collaborative review process allowed the authors to share perspectives, clarify points of ambiguity, and enhance the depth of analysis. Any disagreements, inconsistencies or gaps identified were addressed by revisiting the original sources and refining the information accordingly. The discussions facilitated a more robust understanding of the included studies, which contributed to a more comprehensive and well-rounded approach in interpreting the findings of the scoping review. The insights gathered from this review were critical in ensuring that the final manuscript accurately reflects the research question, inclusion criteria, and objectives of the study.

Finally, the second version of the search strategy included refinements to the table information, incorporating additional insights from the co-creation session. These refinements enhanced the clarity and coherence of the data presented in the tables, ensuring that the key findings were easier to interpret and aligned with the study’s objectives. This version has also updated the categorization of studies, removed any outdated references, and clarified the inclusion/exclusion rationale for some studies.

### 2.7. Data Extraction

In the data extraction process, a scoping review of 58 relevant studies related to validating questionnaires in the area of LL rehabilitation was carried out. The data collection and organization process was carried out through a careful reading of each of the selected studies, together with the use of Mendeley bibliographic management software.

The selected studies were imported into Mendeley and specific categories were created to classify the validating questionnaires found in the reading. In this way, it was possible to effectively organize the information and carry out a detailed analysis of the different questionnaires mentioned in the studies. In such a manner, the 58 studies were carefully examined, extracting and recording the relevant information in tables created in Word. The table contained the following elements: identifying number, bibliographic information, the target population of the study, objective of the study, validation instrument used, the objective of the validation instrument and identified limitations.

During the data extraction process, missing data was identified in specific variables, such as the number of items in the questionnaire, measurement scale, and interpretation of scores, as these were critical elements defined for reporting and analysis. The process for handling missing data followed two steps. (a) Identification: Missing data was flagged during the data extraction process, particularly in key fields that were essential for the analysis and comparison across studies. (b) Handling Strategies: In cases where only a small number of data points were missing, imputation was performed by estimating values based on existing data or patterns. When possible, additional data sources were consulted, such as supplementary articles using the same questionnaire or related sections within the same article, to retrieve the missing information. If missing data was substantial or critical to the analysis and could not be supplemented, the decision was made to exclude the study from the review. However, through supplementary resources, most missing data was resolved, ensuring that the sensitivity of the study remained unaffected.

### 2.8. Selection Process

By adding the key terms in the advanced search provided in the databases, 23,021 studies related to the research questions were identified. Afterwards, the studies obtained were reviewed and 14,160 were removed due to duplication, resulting in 8861 studies. Furthermore, only texts to which the full text was available in English were included, for a total of 5621 studies.

Later, with the studies of interest stored in the reference manager Mendeley, a review was made where inclusion and exclusion criteria presented in Table 1 were applied, where relevant studies were extracted and those that were irrelevant to the research were excluded, resulting in 209 studies.

Starting from the inclusion and exclusion criteria, a second detailed review was made based on the title and abstract, resulting in 81 studies. Then, the inclusion and exclusion criteria were used again for the full-text reading, which yielded 36 results. Finally, 22 additional references found within the studies that also met the criteria were evaluated by means of the full-text reading, resulting in 58 studies as the final result of the search.

### 2.9. Critical Appraisal

A.D.M. conducted a data extraction pilot testing to validate the information in the matrix. The pilot involved the following steps: (a) Selection of articles for pilot testing, ensuring they covered a range of study design, methodologies, and topics relevant to the research questions. (b) Development and testing of the Data Extraction Table, including fields for study characteristics. (c) Filled in the tables with information from the selected articles. After this exercise, the matrix and questions were refined.

### 2.10. Synthesis of Results

Results were synthesized using narrative synthesis to accommodate the diversity of study designs, outcomes, and criteria assessed in the included studies. This method was chosen because it allows for a qualitative summary and comparison of findings, which is suitable given the heterogeneity of the studies (e.g., various validation instruments, populations, and methodologies).

In that way, it provided in a structured, summarized and clear way the key information extracted from each study. This methodology permitted effective organization and visualization of the relevant data on the validating questionnaires used in the area of LL rehabilitation. Table 2 shows the items in each column and their description.

These details were important to understand the context and considerations associated with each validation instrument. Lastly, the results were presented clearly and concisely, using tables and narrative descriptions.

The exploration of heterogeneity was not applicable to this study due to the absence of a meta-analysis or subgroup analysis. The focus of this scoping review was on summarizing the findings across diverse studies rather than statistically assessing differences or causes of heterogeneity.

Similarly, sensitivity analysis was not conducted as it was not relevant to the study design. The emphasis was on qualitative synthesis and descriptive summary rather than on evaluating the robustness of specific quantitative models or results.

## 3. Results

**Figure 2:**
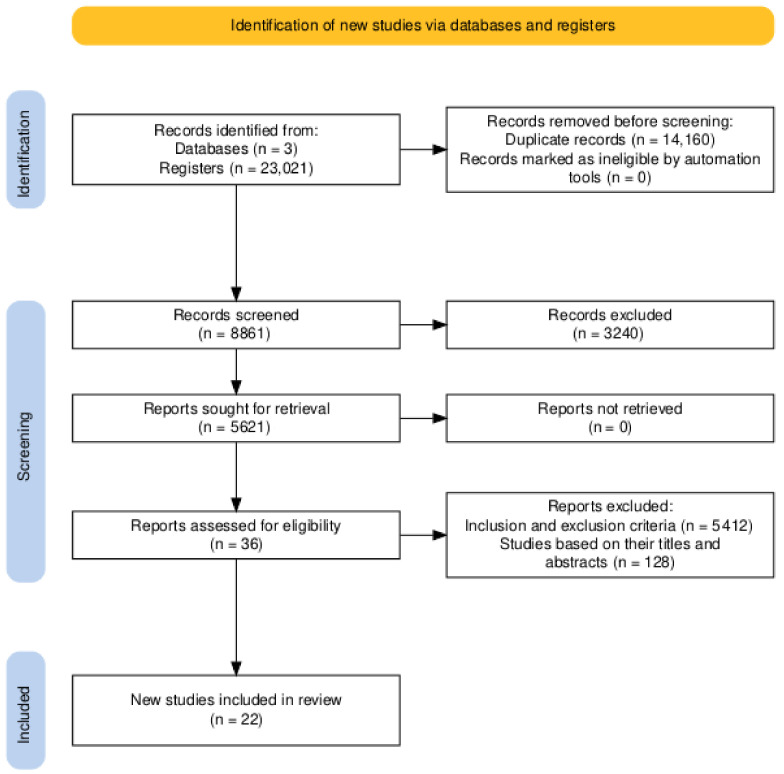
PRISMA flow diagram for the study search and selection process.

This results section uses a flow diagram developed with an online tool to describe the results of the search and selection process, from the number of records identified in the search to the number of studies included in the review (see Figure 2). Additionally, it presents a synthesis of the findings of the scoping review, using tables to classify and organize the selection criteria, the validating instruments and their applications, which allows the information to be grouped, facilitating the understanding and analysis of the data. Moreover, additional information is provided to help understand and contextualize the questionnaires used in the studies.

### 3.1. Evaluation Criteria

The criteria or categories identified in the literature are presented in Table 3, which includes the criterion, the definition of the criterion, the ad hoc questionnaires that have been used by some researchers to assess the criterion and the standardized questionnaires that have been used.

The selection criteria were obtained through a scoping review, and the results obtained were the following: gameplay, enjoyment, usability, expectation of use, motivation, satisfaction, acceptability, user experience, safety, comfort, and immersion. During this process, the different evaluation needs and purposes present in each study were analyzed. In addition, the elements that the questionnaires evaluate concerning the user’s perception were considered.

The purpose of this identification of selection criteria is to be able to determine which questionnaire is most useful in specific applications to assess user perception or experience in systems, devices or exergames utilized in the LL rehabilitation.

Table 4 details psicometric test performed for validating questionnaires, presents general aspects of validity and reliability, and amplify the context and population of studies.

### 3.2. Validation Instruments

In the present scoping review, a total of 23 validated instruments used to evaluate systems, devices or exergames in the area of lower limb (LL) rehabilitation were identified. All of these instruments focus on the user’s perception or experience of the user when interacting with these systems.

Table 5, Table 6, Table 7, Table 8 and Table 9 shows a compilation of the instruments found in the literature, providing important details on their use. It includes the number of items of each instrument, the measurement scale used, the interpretation of the scale and the reference where the questionnaire was proposed.

This table provides an organized overview of the instruments identified, which facilitates comparison and understanding. The details provided allow readers to gain a deeper knowledge of each instrument and its specific characteristics.

### 3.3. Application of Instruments

Table 10, Table 11, Table 12, Table 13 and Table 14 summarizes LL rehabilitation research using systems, devices or exergames in which questionnaires were used as a mechanism for validating progress. The table provides a compilation of citations of relevant research, together with the validation instruments used, the measurement results obtained using these instruments and some additional observations.

Including the citation of each research, allows readers to easily track and reference relevant studies in the field of LL rehabilitation. Additionally, the validation instruments used are mentioned, which offers insight into the tools used to evaluate the progress of each research study.

The measurement results obtained through the validation instruments are presented in the table, providing a synthesis of the relevant quantitative findings in each study and reporting information on the specific tool used to assess improvements in the rehabilitation process.

Also, some additional observations are included in the table, which allows for highlighting relevant aspects or particularities of each research, providing a fuller understanding of the results obtained.

## 4. Discussion

This state-of-the-art review responds to the lack of explicit information identified in the literature regarding selection criteria for technology validation in LL rehabilitation. This article’s primary contribution is identifying specific evaluation criteria for systems/devices/exergames in the area of LL rehabilitation through validation instruments.

Four focus questions guided this review, covering aspects such as identifying evaluation criteria and tools, and analyzing their application and outcomes (see Section 2.2). The results of the scoping review highlight three fundamental elements: (a) Eleven **evaluation criteria** were identified, along with Ad-hoc and standardized instruments supported by the literature to evaluate each criterion. (b) Each selected **validation instruments** was presented with information on its use, measurement scale and interpretation; (c) Significant studies were analyzed, illustrating the **application of instruments and evaluation criteria.**

### 4.1. Evaluation Criteria for LL Rehabilitation Systems Using Validated Questionnaires

The review revealed a lack of systematic guidelines for selecting validation questionnaires for systems, devices, or exergames in LL rehabilitation. Despite the diversity of questionnaires reported in the literature, no standardized selection processes were evident [72]. This study addresses that gap by offering a structured proposal based on the PRISMA methodology, ensuring transparency and reproducibility [12].

This proposal, summarized in Table 3, groups questionnaires by evaluation criteria and aligns with ad-hoc and standardized categories. This framework serves as a valuable resource for researchers, simplifying the process of identifying suitable validation tools for LL rehabilitation studies. Criteria selected are gameplay, enjoyment, usability, Expectation of use, Motivation, Satisfaction, Acceptability, User Experience, Safety, Comfort, and Immersion, supported by studies of the art [73,74]. The scoping review by Nawaz et.al and Tao et.al present several criteria as parameters of their analysis, which align with our proposal [47,75].

The scoping review by Nawaz et al. presents several criteria as parameters of their analysis, which align with our proposal [47].

This proposal is a resource that facilitates the selection process of a specific questionnaire for a future application, since it allows to properly identify the use of validating questionnaires related to the evaluation purpose. This resource is intended to facilitate this selection task in projects that require validation questionnaires for systems/devices/exergames for LL rehabilitation.

Analyzing cost-benefit for ad-hoc and standardized instruments, while standardized tools have undergone rigorous psychometric testing (e.g., content, construct, and criterion validity) making them a broader acceptance in academical and clinical settings, they could have direct cost associated. Conversely, ad hoc questionnaires may be more practical but have limitations as reduced reliability and lack of generalizability. One possible use of this scoping review is that taking an overview of different tools for applications favor a good cost-benefit decision.

### 4.2. Validation Instruments

Table 5, Table 6, Table 7, Table 8 and Table 9, in this review serves as a critical resource, consolidating detailed insights about each identified instrument, including their use or application contexts, number of items, measurement scales, and interpretation guidelines. For example, SUS, with its simplicity and widespread adoption, offers a straightforward 10-item Likert scale to assess usability, making it highly compatible with both clinical and research settings [19,76]. Similarly, GUESS evaluates dimensions such as engagement and challenge in gaming contexts, which are increasingly relevant in gamified rehabilitation systems [27]. By presenting these tools with this useful information, alongside their evaluation criteria (Table 3), the process of selecting appropriate instruments for specific research objectives and compare with similar works is simplified for a researcher.

The increasing incorporation of advanced technologies, such as virtual and augmented reality, into LL rehabilitation underscores the importance of selecting instruments that can evaluate both technical and experiential dimensions. Tools like Unified Theory of Acceptance and Use of Technology (UTAUT) are specifically designed to capture user interaction with cutting-edge technologies [29]. These instruments provide valuable feedback on how technological features influence patient engagement, motivation, and overall therapy outcomes.

Also, there are situations were a questionnaire is adapted from other, taking into account specific populations or conditions. It is the case of Modified QUEST 2.0 Questionnaire that adapts QUEST [45,77]. Other case is the GUESS-18 questionnaire that adapt to only 18 questions the instrument Game User Experience Satisfaction Scale GUESS [27]. The last example is the case of UEQ-S as a short version of User Experience Questionnaire (UEQ) [34].

The comprehensive analysis presented in this review offers researchers actionable insights for integrating the most suitable validation instruments into their projects, ensuring high-quality and impactful rehabilitation solutions.

### 4.3. Application of Instruments

After the selection and filtering process resulted in the identification of references that were considered applicable to systems/devices/exergames in the area of lower limb rehabilitation. In spite of the differences found among the studies regarding the definition of criteria or categories due to their specific evaluation purpose and their application, a unification has been achieved that seeks to provide usefulness for future practical work.

These research studies, detailed in Table 10, Table 11, Table 12, Table 13 and Table 14 becomes a valuable resource in this scoping review and reflect consistency with external evidence, as it shows research that includes validating questionnaires used and that aligns with the established evaluation criteria. These questionnaires address specific needs of LL rehabilitation, such as gameplay, usability, or patient engagement, especially because always this LL systems requires interaction with the user and develop of physical activity. Additionally, as it is confirmed for previous literature reviews, modern systems for LL rehabilitation every time incorporate more technological advances and topics related to virtual reality, augmented reality, and gamification, in enhancing patient motivation and interaction [78]. This references could be the base to other works in Sports or illness rehabilitation in the field of lower limb. While validation questionnaires are particularly valuable during the user interaction phase, rigorous evaluation of technical functionality remains essential, as each system possesses unique technical requirements.

### 4.4. Practical Guidelines for Questionnaire Selection

Researchers can benefit from the following guidelines for questionnaire selection:

(a) Align questionnaires with study goals, e.g., usability, safety, patient satisfaction. (See Table 3).
(b) Select questionnaires according to their specific rehabilitation context (See Table 5, Table 6, Table 7, Table 8 and Table 9).
(c) Ensure relevance to the target population and context with the help of references provided in Tables. See Table 10, Table 11, Table 12, Table 13 and Table 14 as references).
(d) Prioritize user-friendly instruments with proven psychometric properties.
(e) Conduct pilot testing to validate suitability. (See Table 10, Table 11, Table 12, Table 13 and Table 14 as references).
(f) Integrate questionnaires that support real-time feedback and data collection, especially for systems and exergames.
(g) Opt for instruments enabling longitudinal comparisons if required.

These strategies are especially critical in remote rehabilitation settings, where compatibility with telehealth platforms is necessary. In telerehabilitation, some practical implications include ensuring questionnaires can be seamlessly integrated into digital platforms or telehealth systems used in remote rehabilitation; verifying the availability of compatible devices, internet connectivity, and software adaptability; using tools that are user-friendly for both patients and clinicians in remote settings, while still addressing the need for training; and selecting questionnaires with simple formats (e.g., Likert scales or yes/no questions) to minimize technical challenges during virtual interactions.

### 4.5. Limitations

Implementing questionnaires poses several challenges, including a lack of infrastructure and technology, particularly in resource-limited settings. Additionally, the need for trained personnel to administer and interpret complex validation instruments adds another layer of difficulty. When questionnaires are not designed for specific populations or contexts, the absence of proper adaptation and training can lead to misuse or misinterpretation of the data, further complicating their effective implementation.

Existing questionnaires for rehabilitation systems face unique limitations. Many tools lack a specific focus on outcomes related to lower-limb rehabilitation and are mainly useful during the final stages of user interaction. In areas such as device construction, there is a need for the development of new instruments and validation studies. A promising avenue lies in the creation of hybrid tools that combine the rigor of standardized instruments with the adaptability of ad-hoc solutions. Efforts should also include strategies to address contextual barriers, such as designing validation processes for diverse populations and settings.

Cultural and linguistic misalignment of questionnaires presents significant challenges to their implementation. It was noted that some research described validated instruments but mentioned the need to adapt the questionnaires to the specific conditions of the region in which they were applied. This adaptation is crucial to ensure the validity and applicability of the questionnaires in different geographical and cultural contexts. Some recommendations to develop culturally adapted tools involve a systematic process, including translation, back-translation, involvement of bilingual experts, cultural mediation, piloting with target populations, and subsequent revalidation of psychometric properties. Addressing these steps ensures the reliability and validity of adapted questionnaires. Additionally, advancements in technology offer opportunities for digitizing tools, improving accessibility, and enhancing usability in diverse contexts.

In specific, this scoping review identifies several limitations that provide opportunities for improvement in future research. It must be mentioned that although a thorough effort was made to include as much relevant research as possible, the cut-off date for this paper restricted to studies published until May 2023 implies that some research after may not have been considered in the analysis. It is therefore recommended that future reviews take into account more recent research to obtain a more complete and up-to-date picture of the issue.

Within the systematic search, the inclusion and exclusion criteria took into account the lack of accessibility, so some studies were eliminated despite being related to the research topic, which may affect the representation of the results and the completeness of the review. For future work, it is suggested that additional effort be made to gain access to complete information to ensure a more comprehensive review.

### 4.6. Future Directions

Technological advancements are shaping the near future by enabling the creation of more customized and intelligent systems. Artificial intelligence combined with mechatronics systems offers a powerful toolset for the design, development, and validation of rehabilitation systems. Additionally, modern technologies have the potential to positively impact various communities and populations facing disability-related challenges.

Technological advancements will also influence questionnaire validity, particularly in the design, implementation, and validation of questionnaires within specific research contexts.

Advances in data collection have been enhanced by modern tools such as mobile apps, wearable devices, and online platforms, introducing new methods for administering questionnaires. These tools often improve accessibility and scalability but may also pose challenges, such as digital literacy barriers or variations in user interaction, which could affect validity.

Modern advancements involve the integration of various technologies, systems, and devices. In specific contexts of lower limb rehabilitation, advanced technologies such as robotics, virtual reality, and exergames have been widely adopted. In this context, questionnaires designed for these systems must address the criteria analyzed in this paper, including usability and immersion. AI-driven algorithms support adaptive questionnaires that adjust questions based on user responses. This dynamic adaptation and personalization can enhance relevance but pose challenges to traditional validation methods, likely requiring more sophisticated approaches to evaluate consistency and reliability.

## 5. Conclusions

This article has succeeded in identifying criteria for evaluating systems, devices or exergames in the area of LL rehabilitation through validation instruments. A compendium of selection criteria for the application of these instruments is presented, detailing the characteristics of the main instruments and providing relevant research where it has been used.

The systematic analysis conducted in this study has been supported by theory, considering both Ad-hoc and standardized questionnaires, together with the corresponding validation process. This rigorous methodology ensures a thorough and accurate evaluation of the systems and devices used in LL rehabilitation.

Moreover, the tabulated information presented in this article can be used as a reference to establish evaluation criteria, select the most appropriate instrument according to the system to be evaluated or perform a comprehensive review of the state-of-the-art in the field of LL rehabilitation. These tables provides an overview of the instruments and criteria used in previous research, facilitating comparison and analysis of the results.

The selection criteria presented in this study support the process of validating systems/devices/exergames in the area of LL rehabilitation, allowing the identification of relevant questionnaires and their characteristics, as well as comparison with other related work. Additionally, this work has contributed to overcoming some limitations identified in the literature, such as the selection process and the interpretation of questionnaires, and has mapped research using different instruments and analysing different criteria in the field of LL rehabilitation.

While it is acknowledged that there are limitations to this scoping review, such as the exclusion of research due to lack of accessibility, this study has laid the groundwork for future research in this area. Researchers are encouraged to address these limitations and further improve knowledge in the validation of systems and devices for LL rehabilitation.

## Figures and Tables

**Table 1:** Elegibility criteria.

PCC Framework	Criteria
Population	Studies involving patients or users interacting with lower-limb rehabilitation systems. Studies assessing patient experiences or outcomes related to rehabilitation system use.
Concept	Research related to the validation of questionnaires for lower-limb rehabilitation systems. Studies presenting empirical or experimental results concerning rehabilitation systems. Analysis focusing on the design, usability, and effectiveness of rehabilitation systems.
Context	Studies published in conferences, journals, or book chapters. Studies conducted in rehabilitation or assistive technology settings, excluding those strictly related to prosthetic systems. Publications available in English and between January 1998 and May 2023.
Exclusions	Studies unable to provide full-text access. Research solely related to clinical or general-purpose questionnaires unrelated to rehabilitation systems. Studies focusing exclusively on prosthetic devices or unrelated rehabilitation contexts.

**Table 2:** Data extraction items.

Items	Description
Identifier number	Unique number for study identification
Bibliographic information	Title, author, year of publication
Target population	The group of individuals the study focuses on
Study objective	Purpose with the study
Validation instrument	Validation tools used in the study
Instrument purpose	Purpose of validation tools in the study
Limitations	Constraints related to validation instruments

**Table 3:** Criteria for validation in rehabilitation systems.

Criteria	Definition	Ad-Hoc Evaluation Criteria Questionnaires	Standardized Questionnaires
Gameplay	Perception of how the user interacts with a virtual system.		GEQ, SSQ, IPQ, GUESS, GUESS-18, GFQ
Enjoyment	The user’s perception of the physical activity during the test, along with their satisfaction and enjoyment while engaging in physical activity.	[13]	PACES, UEQ, GUESS, GUESS-18, GAME FLOW, IMI, [14]
Usability	User’s perception when interacting with the system regarding its ease of use in an effective and satisfactory way.	Semi-structured interview [15,16]	SUS, QUEST-D, QUEST 2.0, QFQ, TARPP-Q, UEQ
Expectation of use	User’s perceptions and beliefs about the use of a system or product.		CEQ, GUESS, GUESS-18, IPQ
Motivation	User’s perception of his attitude towards the system, which drives him to adopt continuous use of the system.	Semi-structured interview [15]	IMI, GUESS, GUESS-18, PACES, UEQ, GFQ
Satisfaction	User perception in relation to their satisfaction with respect to their needs and according to their expectations.	[17]	QUEST-D, QUEST 2.0, SSQ, CEQ, QFQ, IPQ, GFQ
Acceptability	Willingness of the user to use the system on a regular and continuous basis.		UTAUT, SAR-Q, SUS, TARPP-Q
User experience	Subjective and emotional perception that a user has when interacting with a product or service.	[18], Semi-structured interview [16]	PQ, GUESS, GUESS-18, QFQ, SSQ, IMI, GEQ, TARPP-Q, CEQ, PACES, ITC, GFQ, QFQ, [14]
Safety	Perception of system safety, assessment of risks associated with the use of the technology such as possible side effects or physical damage.		TARPP-Q, SSQ
Comfort	Perception of the user’s physical and emotional comfort during the use of a product or service system.		UEQ
Immersion	User’s perception when feeling the sensation of being completely immersed in a virtual world experience, and its interaction between user and game.		PQ, GEQ, IPQ, ITQ, ITC

**Table 4:** Validity and Reliability Properties of Questionnaires.

Q	Ref.	Validity						Reliability						Population
		✔	Content	✔	Construct	✔	Criterio	✔	Test-Retest	✔	Internal Consistency	✔	Inter-Evaluator	
SUS	[19]	✔	Address dimensions	✔	Correlations SUMI	✗	No ref.	✗	No ref.	✔	Correlations ±0.7−−0.9	✗	No ref.	General population
IMI	[20]	✔	Psychological constructs	✔	FA	✗	No ref.	✗	No ref.	✔	From 0.68 to 0.84 across subscales	✗	No ref.	116 undergraduate students
IMI	[21]	✔	FA	✔	Subscales	✗	No ref.	✗	No ref.	✔	α > 0.90	✗	Not included	226 middle and high school students
CEQ	[22]	✔	Credibility and expectancy	✔	FA	✔	Correlations	✔	Credibility 0.75, expectancy 0.82	✔	0.79<α<0.90	✗	No ref.	Vietnam veterans and spouses (n = 123); GAD (n = 67); PTSD (n = 22)
CEQ	[23]	✔	Perceptions of credibility and expectations	✔	Correlation	✗	No ref.	✗	No ref.	✔	High scores across assessments	✗	No ref.	Subjects in neuro-rehabilitation (n = 17)
SSQ	[24]	✔	Subscale scores	✔	FA	✔	Correlation	✗	No ref.	✔	Consistent symptom scoring	✗	No ref.	Subjects across 9 driving simulator studies (n = 530)
PQ	[25]	✔	Theoretical constructs validated	✔	FA	✔	Correlated presence scores	✗	No ref.	✔	α > 0.80	✗	No ref.	164 participants, VR tasks
PQ	[20]	✔	Theoretical dimensions	✔	FA	✔	High correlations	✔	Stability over repeated measures	✔	α > 0.85	✗	No ref.	Diverse VR participants
GUESS	[26]	✔	Expert reviews	✔	Confirmatory FA	✗	No ref.	✗	No ref.	✔	0.79 < α < 0.90	✗	No ref.	Two studies: n1 = 419, n2 = 197
GUESS	[27]	✔	Iterative expert reviews	✔	Exploratory and confirmatory FA.	✗	No ref.	✗	No ref.	✔	0.79 < α < 0.90	✗	No ref.	Two studies: n1 = 419, n2 = 197
D-QUEST	[28]	✔	Stakeholder panel and testing	✔	Factor composition and correlations	✔	Correlations with PIADS	✔	ICCs: 0.82 (Device), 0.91 (Total)	✔	0.76 < α < 0.82	✗	No ref.	150 Canadian, 243 Dutch mobility users
UTAUT	[29]	✔	Integration of constructs from prior models and expert reviews.	✔	Empirical testing	✔	Predictive validity	✗	No ref.	✔	α>0.7	✗	No ref.	4 organizations with over 250 participants in each.
Semi-structured Interview	[30]	✔	Iterative design process	✔	Capture nuanced participants.	✔	Outcomes and goals.	✗	NA	✔	Structured guide while allowing flexibility	✔	Training interviewers.	Medical educators, researchers, clinical.
GEQ	[31]	✗	Limited evidence of content validity.	✔	Factor structure.	✗	No ref.	✗	Not ref.	✗	No α	✗	NA	Casual and hardcore gamers.
Modified QUEST 2.0	[28]	✔	Expert panel reviews and item analysis.	✔	FA	✔	Correlations.	✔	ICC: 0.82, 0.82, and 0.91 (Total).	✔	α>0.76	✗	NA	Adults using mobility devices.
IPQ	[32]	✔	Expert reviews and refinement	✔	FA	✔	Correlations	✔	Consistent responses	✔	α>0.8	✗	NA	n = 36, aged 19–26, tested VR (Oculus Rift SDK II).
TARPP-Q	[33]	✔	Expert panel Delphi	✔	Exploratory FA	✗	No ref.	✗	No ref.	✔	0.72<α<0.92	✗	NA.	Adult patients aged 18–85 with neurological diseases.
UEQ	[34]	✔	Iterative development and expert reviews	✔	FA	✗	No ref.	✗	No ref.	✔	α>0.7	✗	NA	47 participants, business software users
User Experience Questionnaire (UEQ)	[35]	✔	Scales developed with clear grouping	✔	Correlations	✗	No ref.	✗	No ref.	✔	α>0.7	✗	NA.	n = 219 participants users of YouTube and WhatsApp.
Game User Experience Satisfaction Scale (GUESS-18)	[26]	✔	Expert panel review	✔	Confirmatory FA	✗	No ref.	✗	No ref.	✔	0.722<α<0.890	✗	NA	n = 419 in 2 studies.
Immersive Tendencies Questionnaire (ITQ)	[25]	✔	Iterative development	✔	FA	✗	No ref.	✗	No ref.	✔	α=0.81	✗	Inter-evaluator reliability not applicable.	(n = 24) university students.
Sense of Presence Inventory (ITC)	[36]	✔	Empirical and theoretical frameworks.	✔	FA	✗	No ref	✗	No ref.	✔	0.74<α<0.93	✗	Not applicable.	(n = 600) exposed to films, TV shows, and PC games.
UEQ-S	[34]	✔	Factor loadings	✔	Component analysis	✔	Correlations	✔	Comparing responses	✔	α > 0.8	✗	No ref.	Evaluations of 21 products

Note: No ref: Not explicitly tested or not provided. FA: Factor Analysis. NA: No applicable. Symbols: ✔ = present, ✗ = absent.

**Table 5:** Validation instruments for rehabilitation systems: SUS, IMI, CEQ, SSQ, PQ, GUESS, and D-QUEST.

Instrument	Use	N° Item	Measuring Scale	Interpretation of Score	Reference
System Usability Scale (SUS)	It is a tool to evaluate the usability of a system.	10	Scale from 0 to 100	Usability issues requiring improvement (<70). Good to acceptable usability (70–90). Excellent usability (>90).	[19]
Intrinsic Motivation Inventory (IMI)	It is a self-report questionnaire that assesses the degree to which a person is intrinsically motivated to perform a specific activity.	Original 27 Other versions 18-16	Likert (1 to 7)	“High”, strong intrinsic motivation for a specific activity (≥5). “Moderate”, moderate level of intrinsic motivation (3–5). “Low,” low intrinsic motivation for the activity (≤3).	[20,21]
Credibility and Expectancy Questionnaire (CEQ)	It is a psychometric tool used to measure treatment expectancy and credibility in clinical research and technology use.	6	Likert (1 to 9) or (0% to 100%). Total score (6 to 54).	Ranges vary depending on the research objective. Negative (<13.5). Moderate (13.50–20.25). High (>20).	[22,23]
Simulator Sickness Questionnaire (SSQ)	It is a widely used tool to describe and assess motion sickness before and after virtual reality immersions.	16	Likert (0 to 3). Overall score Max. 48	Higher scores indicate increased severity of both motion sickness and its symptoms. Negligible symptoms (<5). Minimal symptoms (5–10). Significant symptoms (10–15). Worrisome symptoms (15–20). Bad (>20).	[24,37]
Presence Questionnaire (PQ)	It is a tool used to measure presence in virtual environments.	32	Likert (1 to 7)	A higher score indicates a greater experience of presence in the virtual environment. Unacceptable score (0–0.5); acceptable score (0.5–0.75); highly desirable (0.75–1).	[25,38]
Game User Experience Satisfaction Scale (GUESS)	It is a psychometrically validated scale that measures player experience and describes satisfaction with video games.	55	Likert (1 to 7)	The game with the highest score can be considered more satisfactory. It uses Pearson’s correlation.	[26,27]
Quebec User Evaluation of Satisfaction with assistive Technology (D-QUEST)	It is a tool assessing user satisfaction with assistive technology, covering Device and Services components.	12	Likert (1 to 5)	Higher scores typically signify greater satisfaction with assistive technology. The mean of each subscale is predominantly assessed, and it is also possible to establish a cut-off point, depending on the application.	[28]

**Table 6:** Validation instruments for rehabilitation systems: UTAUT, QFQ, Semi-structured interview, PACES, and GEQ.

Instrument	Use	N° Item	Measuring Scale	Interpretation	Reference
Unified Theory of Acceptance and Use of Technology (UTAUT)	It’s a technology acceptance model explaining user intentions and subsequent behavior in using an information system, valuable for predicting technology acceptance and enhancing the design and delivery of technology services.	23	Likert (1 to 7)	A higher score on a dimension is considered to indicate greater acceptance and use of the technology in that specific dimension. They usually determine a cut-off point on the dimensions. Positive acceptance of the technology (>4).	[29]
Qualitative Feedback Questionnaire (QFQ)	It is a tool used to collect qualitative feedback as non-numerical information that measures opinions from an individual perspective.	No default value	Rating based on quality, relevance of comments and qualitative analysis.	Qualitative	[39]
Semi-structured interview	It is a flexible and dynamic resource that allows the interviewer to obtain detailed and in-depth information from the interviewee, who has more opportunities to express fully.	No default value	Qualitative analysis, using content / thematic analysis, to identify common themes and patterns.	Qualitative	[30]
Physical Activity Enjoyment Scale (PACES)	Scale that assesses the degree to which an individual enjoys doing a given physical activity.	18	Likert (1 to 7) Scale 18-126	No specific “good” or “bad” score PACES was found in the search.	[40]
Game Experience Questionnaire (GEQ)	It is a measurement scale designed to assess key attributes of players’ digital gaming experiences.	33	Likert (1 to 7)	Higher scores (≥4) indicate a positive gaming experience and indicate a greater sense of immersion in the game.	[31]

**Table 7:** Validation instruments for rehabilitation systems: Modified QUEST 2.0 questionnaire, IPQ, TARPP-Q, and UEQ.

Instrument	Use	N° Item	Measuring Scale	Interpretation	Reference
Modified QUEST 2.0 questionnaire	It is a widely used tool to assess a patient’s satisfaction with various assistive technologies and has been modified and adapted for different populations, including children.	12	Likert (1 to 5) Max. Scale 12 to 60	A higher score indicates higher satisfaction with the assisted technology. It is used to (a) compare the patient’s score with others, and (b) analyze individual item scores to pinpoint areas of potential challenges for the patient.	[28]
Immersive Presence Questionnaire (IPQ)	To measure the sense of presence experienced in a virtual environment.	14	Likert (1 to 7)	Higher scores indicate a greater sense of presence. It is used to: (a) Compare with the scores of a large group of participants who experienced the same VE, and (b) Analyze individual scores on each IPQ item to identify areas in which the participant experiences more or less presence.	[32]
Technology Assisted Rehabilitation Patient Perception Questionnaire (TARPP-Q)	It is a self-administered, closed-ended questionnaire designed to assess patients’ perception of technology-assisted rehabilitation (TAR)	29	Likert (1 to 4)	The results are analyzed using exploratory factor analysis and their frequency distribution.	[33]
User Experience Questionnaire (UEQ)	It is a survey used to obtain feedback from users of a software or tool.	26	Likert 7 points (−3 to 3)	Positive assessment (values > 0). Negative assessment (values < 0). Rating scales with 5 categories, with the use of a benchmark: (a) Excellent: The evaluated product is among the top 10% of performers. (b) Good: 10% of the benchmark scores are better than the product, 75% of the scores are worse. (c) Above average: the product is located in the 2nd quartile. (d) Below average: the product is located in the 3rd quartile. (e) Poor: the evaluated product is located in the 4rd quartile. Other option: Using importance-performance analysis (IPA).	[34,35,41]

**Table 8:** Validation instruments for rehabilitation systems: GUESS-18, ITQ, and ITC.

Instrument	Use	N° Item	Measuring Scale	Interpretation	Reference
Game User Experience Satisfaction Scale-18 (GUESS-18)	The short version of GUESS, used to evaluate the user experience of video games, measures user satisfaction with various aspects of the game, such as usability, immersion and enjoyment.	18	Likert (1 to 7) Min. scale 9, Max. scale 63	Interpretation of the GUESS-18 score depends on the context and purpose of the assessment. The interpretation of the GUESS-18 score may be based on the mean score, subscale scores, or overall score.	[26]
Immersive Tendencies Questionnaire (ITQ)	Measure individuals’ tendencies to experience presence in virtual environments. Compare ITQ scores between experienced and novice users to assess differences in immersive tendencies between these groups.	29	Likert (1 to 7)	Correlation analysis: It can be performed to assess the relationship between the different dimensions of immersion and perceived control (the higher the score on cognitive immersion, the higher the score on emotional immersion). Analysis of variance: Analysis of variance (ANOVA) can be used to assess differences in immersive tendencies between different groups of participants. In some cases, cut-off points or thresholds can be established for ITQ scores that indicate low, medium, or high levels of immersion and perceived control. These cutoff points may vary.	[25]
Sense of Presence Inventory (ITC)	Developed to assess users’ experiences with the media, without reference to objective System parameters. It has been based on previous research on the determinants of presence and current self-report measures.	44	Likert (1 to 5)	Higher scores indicate a greater sense of presence in the experience. There is no fixed scoring scale for this questionnaire. Instead, researchers can adapt the scales as needed for their specific study. Scores can be interpreted relatively, comparing the results of the experimental group with those of the control group or with the results of other previous studies.	[36]

**Table 9:** Validation instruments for rehabilitation systems: Ad-Hoc Questionnaire, GFQ, UEQ-S, Questionnaire of Gerling et al. [14].

Instrument	Use	N° Item	Measuring Scale	Interpretation	Reference
Ad-Hoc Questionnaire	Questionnaire that is created for a specific purpose. It is not standardized but is customized to fit the needs.	There is no set value	There is no set scale	Determined according to the needs of the study.	[42]
GameFlow questionnaire (GFQ)	It is an instrument used to assess the perceived usefulness of a game, which is operationalized as enjoyment, is used to assess a player’s level of enjoyment of the game, which facilitates improvements in the implementation and design of a game.	18	Likert 7 points	In one study, it was interpreted with the mean and compared with other systems by factors. The average knowledge improvement is approximately 5 points, the higher the average score, the more effective the factor evaluated.	[43]
Short version User Experience Questionnaire (UEQ-S)	The short version of the UEQ, UEQ-S, is a valid tool to evaluate the subjective opinion of users towards the user experience provided by a product.	12	Likert 7 points	Evaluated with the mean, or using the same methods as the UEQ.	[34]
Questionnaire of Gerling et al.	Developed to assess the experience and performance of seniors using exergames.	10	Likert 5 points (0 to 4)	The search did not find a cut-off point to assess whether their experience was good or bad, but comparisons, descriptive statistics, inferential statistics (t-test axis) and qualitative analysis are used to interpret the results. Comparisons, descriptive statistics, inferential statistics (e.g., *t*-test) and qualitative analysis are used to interpret the results.	[14]

**Table 10:** Lower limb rehabilitation investigations using validating questionnaires. Part 1.

Reference	Instrument Used	Measurement Result
[18]	Ad-Hoc Questionnaire	60% of the population evaluated reported difficulties with technology use. Non-standardized questionnaire, it does not allow comparisons with other studies.
[44]	IMI	The mean value of interest and enjoyment was 4.593/7.000. It was considered a good result, indicating that the user was motivated to use PedaleoVR. Limitation: Inability to distinguish between intrinsic and extrinsic motivation.
	CEQ	Participants express a belief in the rehabilitative benefits of cycling with PedaleoVR, (18,300 ± 5595). In addition, they hold moderate expectations (15,050 ± 6004) regarding the improvement of their physical function through this intervention.
	SSQ	None of the subscales, surpass 20 points, indicating the VR cycling platform doesn’t induce significant negative effects.
	PQ	The patients’ overall QTS score (71.000/108.000 ± 23.225) suggests a moderately high level of satisfaction with the EVE. Improving the virtual reality platform with photorealistic graphics may enhance the user’s perception of actionability.
	GUESS-18	The score obtained in the “Social Connectedness” subscale (5500/7000 ± 1949) is particularly remarkable. The satisfaction subscale is positive and moderately high, it can be interpreted similarly to the values of the intrinsic motivations subscale obtained with patients with LBP. The overall score of all patients is 51.647 out of 63, which confirms that the participants were always satisfied with the system used.
	SUS	SUS results in DLL patients (80.375 ± 15.558) indicate that the degree of use of the PedaleoVR is very good. This measure did not seem sufficient in the case of older participants, who, in general, showed high confidence in the system during the experimental tests, so the score obtained in the EUE was almost 12 points lower (68.472 ± 18.145).
[45]	QUEST 2.0	On the devices subscale, participants’ ability levels ranged from 7.46 logits (from −2.35 to 5.11, where the mean measure of non-extreme individuals was 0.69), and item difficulty estimates ranged from 0.73 logits (from −0.33 to 0.40).
[28]	QUEST 2.0	Although the majority of respondents reported always being satisfied with the devices, a considerable percentage (19%) expressed specific concerns and general dissatisfaction.
[46]	D-QUEST	Mean satisfaction with the exoskeleton was 3.7 ± 0.4 (total D-QUEST), 3.5 ± 0.4 (assistive device subscale) and 4.2 ± 0.5 (service subscale). “Weight”, “Efficacy”, “Ease of use” and “Safety” were most frequently scored as dissatisfied (D-QUEST item score < 3) and—at the same time—indicated as important.
	SUS	The usability of the exoskeleton was scored with a median of 72.5 [52.5–95.0]. Two SUS items had a median score of less than 3, indicating low usability.
[15]	SUS	The score obtained (58.3 ± 16.5) revealed a level of ease of use between “acceptable” and “good”.
[47]	SUS	Usability was considered good when the value ranged between 87 and 89 on the 0–100 scale, older adults had a better experience with the SilverFit exergame (SUS = 87.0 ± 11.1).
[48]	UTAUT	The PAE category showed a very positive response. Thus, the participants showed a positive attitude and a favorable acceptance of the functioning of the system.

**Table 11:** Lower limb rehabilitation investigations using validating questionnaires. Part 2.

Reference	Instrument Used	Measurement Result
[49]	Ad-hoc	Patients’ opinions were taken into account, and patients made several suggestions for improving teletherapy. Patients showed moderate to high acceptance rates for the teletreatment, with mean scores ranging from 6.1 to 9.3 on the 11-point Likert-type scale.
[50]	Ad-hoc	The mean score of the questionnaires for the MCS and PCV modalities is shown, and a significant difference was observed according to the Wilcoxon test (Z = −5.34, *p* = 0.000). The questionnaires indicated that MCS was easier to accept by the subjects.
[51]	QUEST 2.0	The participants’ perception of the device according to the modified QUEST 2.0 scale was acceptable, and all participants were able to wear the device comfortably during all protocols. Dimensions, weight, safety and comfort were the most highly rated aspects. The item with the lowest score was related to the effectiveness of the device in solving the participants’ problems (mean score 2.4 ± 0.5). According to the participants’ selection, the best features of the exoskeleton were its comfort, safety and ease of use.
[52]	GEQ	For the GEQ questionnaire, Kina’s results were superior, showing an adequate cohesion of the different aspects of the Exogames and a positive evaluation of the system by the users.
	SUS	Participants rated usability with 69 (66, 79), which is well within the range of acceptability.
	PACES	PACES scores 110 (108, 112), which means pleasant. The latest evaluation suggests that Exogames can be used as a virtual reality game for different purposes, including rehabilitation.
[13]	Ad-hoc	All subjects in the EG group and 75.8% in the Control group evaluated the exercise program as pleasant, quite pleasant or very pleasant.
[53]	IMI	Shows that patients expressed significant preferences for the two-player mode in all three categories (fun and interest: *p* < 0.01; perceived competence: *p* < 0.01; effort and importance: *p* < 0.001) of the IMI assessed.
[54]	UEQ-S	The practical quality value (1.63 ± 0.85), the hedonic quality value (1.75 ± 0.86) and the total value (1.69 ± 0.86) of 20 patients indicate that the system provides an excellent user experience, which improves patients’ willingness and compliance for active exercise.

**Table 12:** Lower limb rehabilitation investigations using validating questionnaires. Part 3.

Reference	Instrument Used	Measurement Result
[55]	[14]	Most participants expressed that it was fun to use the sensor-based fitness training technology (median 4). Participants felt safe while using the exercise technology, did not experience fear of falling, never lost their balance while exercising (median 0), and did not need help to keep their balance while performing the exercises (median 3). For most participants, the balance exercises were not difficult to perform and did not go too fast.
[56]	[14]	Most participants strongly agreed that they had fun while exercising, experiencing no problems or safety concerns (mean score > 3.5). The feedback sensor helped most participants learn the exercises (mean score = 3.4). Participants moderately agreed that the form and design of the technology were optimal (mean score = 3.1). Most participants disagreed that the exercises were too fast or required support to maintain balance (mean score < 0.5). Participants moderately disagreed that the movements were difficult to perform (mean score = 1.0) showing the descriptive results of the user experience questionnaire in mean, standard deviation, median and range.
[57]	SUS	Obtained a score above the cut-off point (68). The lowest score obtained was 70, while the highest score was 100, and the mean was 81.5. This score indicates a good result in the SUS questionnaire. The results showed a high acceptance in terms of usability of the FRED game among the participants of the study group.
	Ad-hoc	Question 1 all 20 participants in the study group responded positively on all days except days 1 and 2, in which case there was a negative response (10%-2 participants-and 5%-1 participant-respectively). Question 2 all 20 participants in the study group responded positively on all days except days 1 and 2, in which case there was a negative response (20%-4 participants-and 5%-1 participant-respectively). Showed that it can motivate frail elderly people to exercise, as it is a game that they like and that is motivating to improve their physical condition.
[58]	UTAUT2	The results showed that older people perceived relatively high vulnerability (3.21 out of 5) and severity (3.63) concerning difficulties in self-care and independent living, and had high intentions (Behavioral Intention = 4.08) to use our system in the future. They thought our system was very useful (Perception of usefulness = 4.43), positive (Attitude = 4.29), entertaining (Hedonic motivation = 3.82), and low privacy risk (Perception of privacy risk = 1.18). Interestingly, although older people had some confidence in their abilities to use this system to improve their health status (Self-efficacy = 3.75), the expected effort (3.07) and response cost (2.72) were considerably high.

**Table 13:** Lower limb rehabilitation investigations using validating questionnaires. Part 4.

Reference	Instrument Used	Measurement Result
[59]	Semi-structured interview	Each item was evaluated qualitatively. The results revealed that the motivation to play could be kept high when the scores of competitors with similar skills were displayed on a high-score board. Our results indicated that, due to a lack of knowledge and experience, OAs showed a negative attitude and were hesitant to use a new technology. Other studies have found other barriers to the use of technology, such as age-related vision and hearing loss and fine motor impairment.
[60]	SUS	Both the manikin and the mobile application obtained SUS scores above 70/100, indicating that both are usable. (performed a statistical analysis of variance (ANOVA) on the usability data) there were no usability differences between the manikin user interface and the mobile app when used to display exercises for the lumbar spine. Similar results were obtained.
[61]	SUS	The results show that the means of users’ overall satisfaction with all heuristic items, from H1 to H10, are between 4.20 and 4.47 point. This interval means that users’ ratings of the heuristic usability factors are between “satisfied” and “very satisfied”. showed that there was a strong correlation between the interface design and the usability of the exergame system. With an improved interface, users were better able to interact with the system, and the usability of the entire system, including both the device and the system itself, improved. As a result, the proposed usability model could be used to evaluate other exergame systems.
[62]	IMI	The results show a high level of agreement in the scores given by the physical therapists in the 6 subscales evaluated. Perceived competence and usefulness of the tool obtained the highest scores, indicating that the physical therapists feel capable and find the tool useful for rehabilitation. Interest and enjoyment in using the tool were also high. However, it was noted that some practitioners would require more encouragement to adopt the new tool, and the pressure and stress associated with using the tool were rated as low indicating that practitioners believe they would not experience moments of pressure and stress when using the tool. The professionals were the ones who tested the system.
	SUS	The results are also positive, with 60% considering the application as excellent, 30% as good, 5% as acceptable and the remaining 5% as deficient.
[16]	Semi-structured interview	The results showed that for people with good physical ability, the games may be too easy to perform. Another usability problem reported was the small size of the balance board, which raised concerns about possible falls. The results provide valuable information on the feasibility of video games in the context of rehabilitation and physical activity promotion Qualitative results.
[63]	UEQ	In the evaluation with physiotherapists, all scales present values above 1.5, reflecting acceptance.

**Table 14:** Lower limb rehabilitation investigations using validating questionnaires. Part 5.

Reference	Instrument Used	Measurement Result
[64]	Ad-hoc	Initial impressions of Wii Fit were mostly positive at the beginning of the study (e.g., Wii Fit would be a fun way to engage in physical activity, increase physical activity levels and improve fitness level). However, some participants expressed concerns about learning to use the technology, and others felt some of the games moved too fast or the board was too small.
[65]	SUS	The average System Usability Scale scores for video and avatar feedback were 39.3 ± 29.5 and 28 ± 18.8, respectively out of a total of 100 points. There was no significant difference for System Usability Scale scores between video and avatar feedback (t9 = 1.02, *p* = 0.33). The System Usability Scale score was low due to the passive nature of this visual feedback system and the occasional tracking errors that affected the avatar display.
[66]	QFQ	The proportion of participants choosing positive, neutral, and negative responses in the QFQ-Questionnaire will be reported to inform about the acceptability of the protocol. Adherence will be assessed by the proportion of participants who completed the study compared with the total number who completed baseline assessments. For all analyses, a significance of 5% will be considered.
[67]	Ad-hoc	Despite cupping therapy being reported as ‘uncomfortable’ it is Acceptable. 100% of participants said that they would receive cupping again in the future. They receive some opinions about pain, ROM, muscle tension, and relaxation.
[68]	Ad-hoc	Seven subjects who used Ligaflex found it more stable. Except for subject 4, all found Balleta the most uncomfortable and the least stable.
[69]	UEQ	The rate for user acceptance is 81.09% and 80.77% on average among participants and human trainers, respectively.
[70]	PACES	The mean (SD) physical activity enjoyment (PACES) scores did not change over time (baseline 71.2 vs. 8 weeks 69.2, *p* = 0.45) nor differ between the groups (*p* = 0.07).
	SUS	The Physitrack app was reported to be highly usable by all participants (mean score 86, SD 10). Participants most strongly agreed that they felt confident using it and that most people would learn to use the system very quickly. Participants also strongly disagreed that the system was cumbersome and unnecessarily complex.
[71]	SUS	The results showed that the video game-based rehabilitation method designed in this study performed equally well as the traditional method in subjects with prior rehabilitation experience except for the degree of fun, which was higher for the game-based method. Compared to traditional rehabilitation, video game-based rehabilitation was accepted to a greater extent in elderly subjects without prior rehabilitation experience; it was more likely to trigger them to perform rehabilitation, reduce their resistance to long-term rehabilitation, and increase their rehabilitation intention.
	Semi-structured interview	The results of the qualitative analysis revealed that the somatosensory interactive game developed in this study was easy to understand, with approachable cartoon characters easily identifiable. The real-time feedback and scoring system provided by the game allowed elderly users to clearly understand their performance. Meantime, the game-based rehabilitation method helped the users to develop their concentration and improve their rehabilitation motivation and interest.

## Data Availability

Not applicable.

## Supplementary Materials

The following supporting information can be downloaded at: https://www.mdpi.com/article/10.3390/sports13010004/s1. The completed checklist for the Preferred Reporting Items for Systematic Reviews and Meta-Analyses for Scoping Reviews (PRISMA-ScR) criteria is available as supplementary material for this paper [12].

## Abbreviations

The following abbreviations are used in this manuscript:

SUS	System Usability Scale
IMI	the Intrinsic Motivation Inventory
CEQ	Credibility and Expectancy Questionnaire
SSQ	Simulator Sickness Questionnaire
PQ	Presence Questionnaire
GUESS	Game User Experience Satisfaction Scale
D-QUEST	Quebec User Evaluation of Satisfaction with assistive Technology
UTAUT	Unified Theory of Acceptance and Use of Technology
QFQ	Qualitative Feedback Questionnaire
PACES	Physical Activity Enjoyment Scale
GEQ	Game Experience Questionnaire
IPQ	Igroup Presence Questionnaire
TARPP-Q	Technology Assisted Rehabilitation Patient Perception Questionnaire
UEQ	User Experience Questionnaire
ITQ	Immersive Tendencies Questionnaire
ITC	Sense of Presence Inventory
GFQ	GameFlow questionnaire
UEQ-S	short version User Experience Questionnaire
